# Perianal abscesses due to ingested foreign bodies

**DOI:** 10.4103/0974-2700.70769

**Published:** 2010

**Authors:** Mbarek Doublali, Ali Chouaib, Mohammed Jamal Elfassi, Mly Hassan Farih, Bachir Benjelloun, Younes Agouri, F Z Zahid, A Louchi

**Affiliations:** Department of Urology, University Hospital Center, Fez, Morocco; 1 Department of Urology and Surgery, University Hospital Center, Fez, Morocco

**Keywords:** Foreign body impaction, perianal abscess, swallowed bone

## Abstract

The clinical presentation of perianal abscesses due to foreign bodies (FBs) impacted in the anal canal mimics common causes of acute anal pain. The diagnosis can be established by digital rectal examination and/or proctoscopy, but may miss the presence of an FB. Incision and drainage of the abscess, along with removal of the FB, results in immediate pain relief and cure. Impacted FB must not be overlooked as an unusual cause of perianal abscess. One case of perianal abscesses due to FB impacted in the anal canal is reported.

## INTRODUCTION

The anal canal is an unusual site of foreign body (FB) impaction. In the current literature, only six cases have been reported. Ingestion of FB is a common surgical problem. Most of the times, the ingested body passes through the gastrointestinal (GI) tract uneventfully. However, few cases have been reported in the literature with peritonitis or intra-abdominal abscesses, secondary to perforation of the GI tract. Most common sites of impaction and perforation include the appendix, cecum and the terminal ileum.[1,2] Anal stenosis or spastic anal sphincter may predispose to FB impaction in the perianal canal. Digital examination (DE) and proctoscopy can establish the diagnosis of the abscess, but do not necessarily demonstrate the presence of the impacted FB.[3] Early incision, drainage and adequate exploration of the abscess cavity can help identify and remove the FB.

We report one case of impacted FB in the anal canal, which resulted in perianal abscess formation.

## CASE REPORT

**Figure 1:**
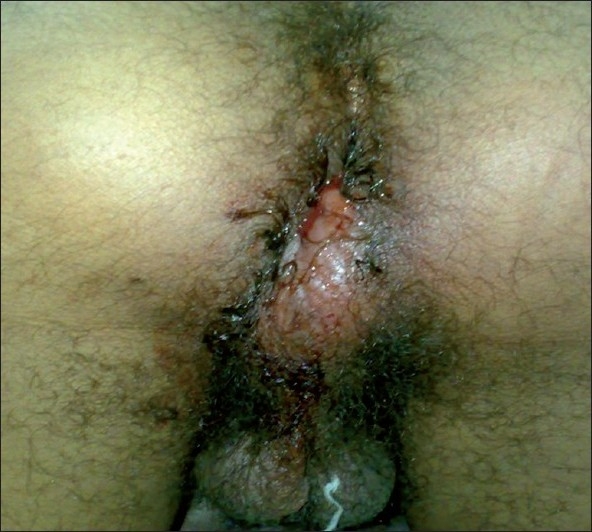
Localization of the perianal abscess

A 46-year-old man gave no significant past history, presented to the department of emergency with a 6-day history of severe rectal pain, fever, perianal swelling and bloody discharge. On examination, he was in obvious distress, being unable to sit without experiencing excruciating rectal pain. He had a correct blood pressure and a normal pulse and respiratory rate. The body temperature (axillary) was 38.5°C. He had a perianal swelling at 6 o’clock position [Figure 1].

**Figure 2:**
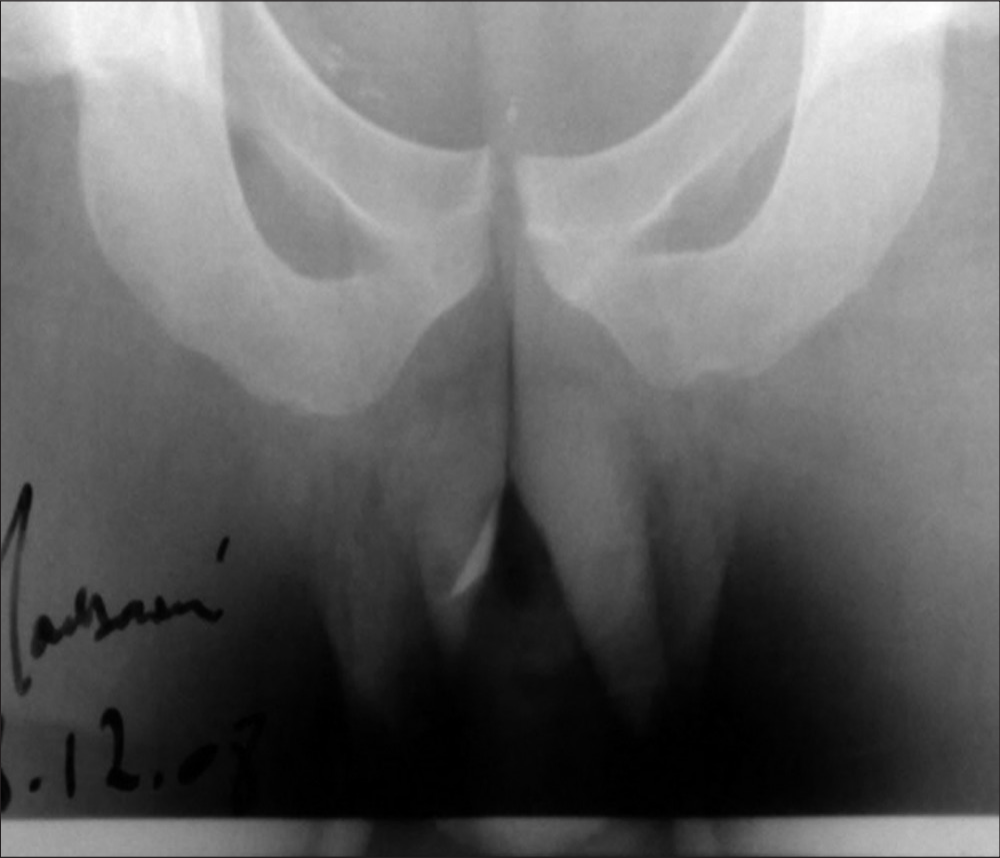
Abdominal X-ray objectified the foreign bodies

**Figure 3:**
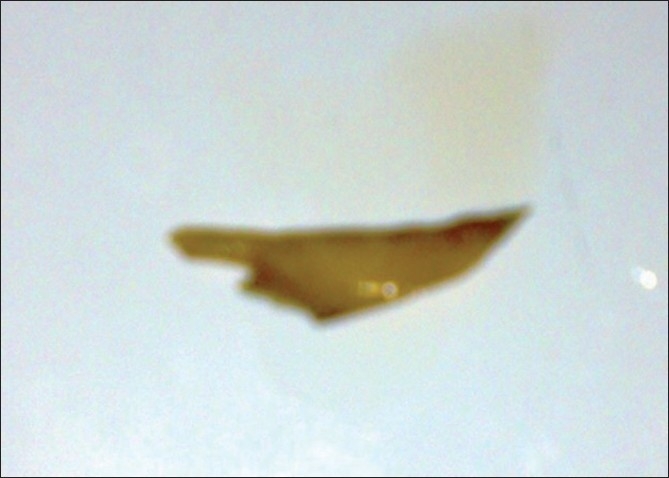
FB (fragment of lamb bone)

Digital rectal examination revealed a perianal abscess and identified the presence of an FB encrusted in the walls of the anal canal [Figure 2]. Abdominal and systemic examination was normal. Abdominal X-ray confirmed the presence of an FB and its morphology [Figure 3]. The abscess was drained under spinal anesthesia by a large incision; 50 ml of pus was removed. Intraoperatively, an FB (fragment of lamb bone) measuring 10 by 30 mm was seen inside the perianal abscess cavity and a small opening connecting to the anal canal could be identified. We treated the case as an anal fistula and fistuolotomy was performed. The wound was packed with iodophor gauze. The gauze was removed after 24 hours and the patient was instructed to take sit baths three times a day and after bowel movements. Postoperatively, the patient was discharged from the hospital in a stable condition 3 days later. Analgesics and stool softeners were prescribed to relieve pain and prevent constipation. The recovery was uneventful 18 months later. He had no recurrence, with total conservation of the anal continence. On further questioning, the patient recalled eating meat of the lamb 8 days prior to onset of pain. He denied being under the influence of alcohol at the time but had a habit of not chewing food thoroughly.

## DISCUSSION

Impacted FB in the anal canal is an unusual cause of perianal abscess formation. Only few cases have been reported in the literature so far.[3,4] Bones are often sharp objects, which can get lodged in the aerodigestive tract and cause complications. Although this is rare (about 1-3%), the associated complications are potentially catastrophic including cervical abscess, mediastinitis, esophago-carotid fistula and perforated bowel. In our case, a lamb bone ingested by the patient was impacted in the perianal space. Probably, the force exerted by the anal sphincter and by the evacuated fecal matter during defecation resulted in this sharp object being pushed through the anal wall with its pointed end leading into the perianal tissue. Subsequently, infection resulted in abscess formation. Prolonged history of pain can be associated with prolonged and neglected impaction, which leads to abscess formation.[5] Risk factors predisposing to impacted ingested FB include the presence of dentures which may decrease the sensitivity of the palate, previous anal surgery complicated by anal stenosis and alcohol intoxication.[6]

The diagnosis of a perianal abscess can be made by careful DE and/or proctoscopy.[7] However, DE often fails to reveal the presence of an FB in the abscess cavity. Sometimes, X-ray of abdomen before DE can help to prevent accidental injury to the surgeon from sharp objects. However, it is not indicated in a majority of cases of perianal abscess. Pelvic and abdominal X-ray can occasionally help in localizing the FB and also rule out intestinal perforation. The lateral films of pelvis orient whether the FB is high or low lying but low FB will not be obvious hiding behind the pelvic bone, on the lateral abdominal X-ray.

Adequate incision and drainage with removal of the FB resulted in immediate pain relief and patient satisfaction with minimal morbidity. Intraoperatively, careful examination and adequate exposure helped to identify and remove the impacted FB. Proctoscopy may be needed to assess the extent of the local injury, but does not necessarily visualize the FB due to the impaction. We did not perform other investigations, such as computed tomography or magnetic resonance imaging in the acute setting of a perianal abscess; however, if any doubt exists, these diagnostic tests can help establish the presence of FB.[8]

## CONCLUSION

Impacted ingested FB is a very rare cause of perianal abscess. Predisposing factors include long history of perianal pain, use of dentures or alcohol, history of anal stenosis and/or previous anal surgery and should raise suspicions of impacted FB. Examination under anesthesia and good exposure of abscess cavity are the key maneuvers to identify and subsequently remove the impacted FB.
